# Effectiveness of Lee Silverman Voice Treatment (LSVT)-BIG for Neurological Diseases Other than Parkinson’s Disease: Mini Review

**DOI:** 10.3390/brainsci15040367

**Published:** 2025-03-31

**Authors:** Changyeon Won, Woohyuk Jang, Sunwook Park

**Affiliations:** 1 Department of Occupational Therapy, Graduate School, Kangwon National University, Samcheok 25949, Republic of Korea; wohyon@naver.com; 2 Department of Occupational Therapy, Kangwon National University, Samcheok 25949, Republic of Korea; otjang@kangwon.ac.kr; 3 Department of Physical Therapy, Kangwon National University, Samcheok 25949, Republic of Korea

**Keywords:** review, neurological disease, LSVT-BIG, intervention, rehabilitation

## Abstract

**Background:** Lee Silverman Voice Treatment-BIG (LB) was developed for Parkinson’s disease patients to improve patients’ movement amplitude and accuracy through large movements and enhance movements through self-awareness and recalibration. This study aimed to review studies on LB for neurological diseases other than Parkinson’s disease and examine its potential as an intervention tool. **Method:** The main search databases included Google Scholar, PubMed, and ScienceDirect. ‘Neurological disease’, ‘LSVT-BIG’, ‘Treatment or Rehabilitation’, ‘Intervention’, and ‘Therapy’ were used as search keywords until December 2024, and eight articles were finally selected. **Results:** As a result of analyzing eight studies, there were four studies on stroke (all conducted by occupational therapists) and four studies on other diseases, including two studies on progressive supranuclear palsy, one study on idiopathic normal pressure hydrocephalus, and one study on Huntington’s disease (all conducted by physical therapists). **Conclusions:** LB had a positive effect on improving physical function and overall motor control in patients with neurological diseases other than Parkinson’s disease, indicating its potential as an intervention tool. In the future, studies that have high-level evidence-based study designs and complement small sample sizes are needed to demonstrate the effectiveness of LB.

## 1. Introduction

Neurological diseases are a term referring to a wide range of damage caused by various injuries, degeneration, inflammation, and infections of the central nervous system [1,2]. Neurological diseases include stroke, Traumatic Brain Injury (TBI), Parkinson’s disease, Alzheimer’s disease, and other diseases such as dementia, migraine, epilepsy, etc. [3]. Depending on the site of damage and diagnosis, neurological diseases cause significant disruption in daily life due to various problems in movement, sensation, cognition, and mental functions, and the prognosis is even worse in the case of progressive diseases [4]. These neurological diseases are increasing along with the increase in the elderly population [5], and in addition to physical and mental problems, the increase in social costs is emerging as a national problem [3,6].

Meanwhile, several interventions have been attempted by various experts for the rehabilitation of these neurological diseases [7,8,9,10,11,12,13,14]. Therefore, a variety of intervention methods have been introduced: intervention combining occupational therapy and motor learning (gait for stroke patients) [8], therapist-assisted locomotor training (gait for stroke patients) [9], supervised exercise therapy (cardiorespiratory fitness for TBI patients) [10], overground walking (gait for stroke patients) [11], aerobic exercise (fatigue for multiple sclerosis patients) [12], repetitive training of many fixed movements (hand function, strength, and mobility for stroke patients) [13], and virtual reality (gait for stroke patients) [14]. Various efforts have been made to address these neurological diseases and have reported success.

Recently, a treatment called Lee Silverman Voice Treatment (LSVT) has been in the spotlight [15]. Lee Silverman Voice Treatment (LSVT) was developed as an approach for speech treatment for Parkinson’s disease patients by Ramig and Bonitati in 1987 [16]. LSVT is currently divided into LOUD and BIG, and LSVT-LOUD is used as a speech treatment protocol to promote functional communication [16]. On the other hand, LSVT-BIG (LB) was developed based on LOUD with the goal of improving amplitude to improve problems such as bradykinesia/hypokinesia [17]. LB aims to improve the amplitude and accuracy of patient movements through standardized large movements of the entire body and to enhance rapid movements [18]. The characteristics of LB are focused on intensive training of large-amplitude movements [19] and emphasize high effort and intensive treatment [20]. For this reason, LB enables the recovery of normal movement amplitude through the recalibration of perception for movement execution [21].

The composition of LB program is as follows: (1) Daily Exercise, (2) Functional Component Movements, (3) Hierarchy Tasks, (4) BIG Walking, and (5) Homework Practice [15]. First, daily exercise basically refers to the repetition of seven full-body movements (sitting position: two, standing position: five). Functional component movements refer to the designation/repetition of up to five detailed movements (e.g., sit to stand) that the subject wants to perform more smoothly in daily life. Hierarchy tasks are divided into complex multilevel tasks with higher difficulty by adjusting the amplitude and effort, for the subject to be able to move smoothly in various and special environments, and are repeated step by step (e.g., basic bathroom skills versus going out to dinner). BIG walking is training with large strides and consists of various moving distances and time restrictions. Finally, homework practice is a program that can be done at home, and the training time varies depending on the treatment status on the day. Meanwhile, studies are being published on a modified LB that streamlines the standard protocol of four times a week for 4 weeks [22,23]. Five sessions per week for 2 weeks [22] or 4 weeks, two with a therapist and two independently at home [23], have been reported to be effective for Parkinson’s patients. Modified LB has also been reported as an effective intervention for Parkinson’s patients. The detailed composition for standard LB is as follows (Table 1).

## 2. Methods

**Figure 1:**
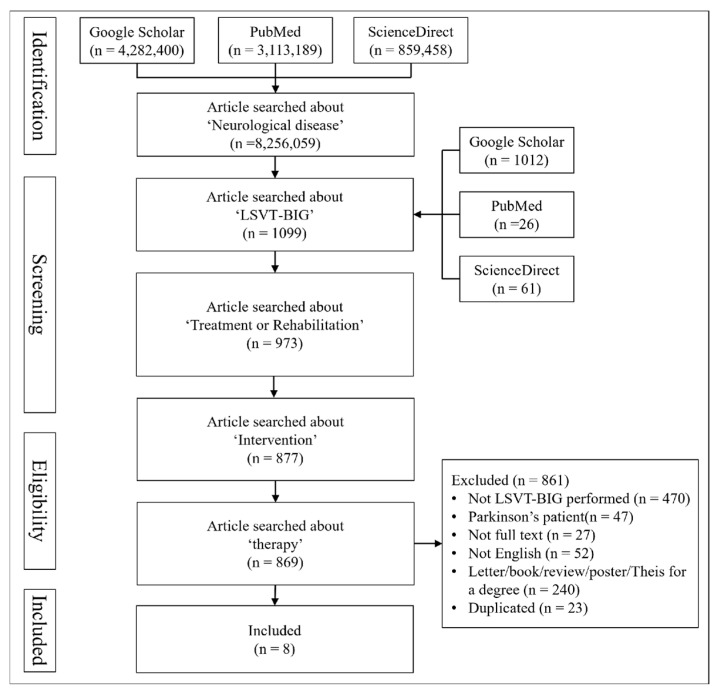
Flowchart of Inclusion process.

This study conducted a literature review on LB studies for stroke patients and patients with neurological diseases. Data were collected as of December 2024, and the databases including ‘Google Scholar’, ‘PubMed’, and ‘ScienceDirect’ were used. The keywords used for the search were as follows: ‘Neurological disease’, ‘LSVT-BIG’, ‘Treatment or Rehabilitation’, ‘Intervention’, and ‘Therapy’. A total of eight articles were selected after excluding studies that met the exclusion criteria and were retrieved multiple times (Table 2) (Figure 1).

## 3. Results

There was a total of eight studies of LB interventions for neurological diseases other than Parkinson’s disease. Four studies were conducted for stroke [27,28,29,30], and four studies were conducted for other neurological diseases [31,32,33,34] (two studies on Progressive Supranuclear Palsy (PSP), one study on Idiopathic Normal Pressure Hydrocephalus (INPH), and one study on Huntington’s disease).

All studies on stroke were conducted by occupational therapists, and all other studies were conducted by physical therapists. As for LB study design, case reports conducted by physical therapists [31,32,33,34] were the most common, with four studies, followed by two case studies conducted by occupational therapists [27,29], and one study each with a single subject design [28] and a waitlist crossover design [30]. As for the subject’s inpatient/outpatient status, there were seven studies targeting outpatients [27,28,30,31,32,33,34] and one study targeting inpatients [29].

### 3.1. Four LB Studies on Stroke Patients

Proffitt et al. (2018) conducted a case study to investigate the possibility of intervention through LB for one outpatient with stroke (56-year-old female, cerebral infarction, left hemiplegia, onset period 29 months, premorbid dominant hand: left hand) [27].

The LB intervention was conducted by an occupational therapist. On days with LB, four trainings (daily exercises, functional component movements, hierarchy tasks, and BIG walking) were conducted for a total of 16 sessions. On days without outpatient treatment, homework practice was conducted. Homework practice was conducted with ‘Mystic Isle’, a virtual reality (VR) game that is not only good for high adherence and motivation, but also easy to induce large amplitude. No intervention other than LB was conducted during the entire training period.

The evaluation was conducted three times in total: pre-intervention, post-intervention, and 6 months after the intervention. In a comparison of pre- and post-intervention, improvements were shown in the spasticity of the elbow flexors in the Modified Ashworth Scale (MAS) that evaluates the degree of spasticity (pre: 3, post: 2) and the performance time of the Wolf Motor Function Test (WMFT) that evaluates the function of the upper extremity (pre: 17.42 s, post: 7.94 s, total 45% reduction). In the Canadian Occupational Performance Measure (COPM, total score: 10) that evaluates occupational performance, performance improved by 4.1 points (pre: 2.4, post: 6.5) and satisfaction improved by 5 points (pre: 1.0, post: 6.0). In the Performance Assessment of Self-Care Skills (PASS) that evaluates activities of daily living, independence improved by 0.25 points (pre: 2.75, post: 2.95), safety improved by 0.2 points (pre: 2.8, post: 3.0), and adequacy improved by 0.2 points (pre: 2.0, post: 2.2), respectively. Finally, the Stroke-Specific Quality of Life scale (SS-QOL, total score: 245) that evaluates quality of life improved by 23 points (pre: 195, post: 218). Additionally, the follow-up evaluation 6 months after the end of the intervention showed a score that was slightly decreased compared to immediately after the end but improved compared to before the intervention. Based on this, the LB effect on stroke patients was demonstrated for the first time. In addition, the potential of ‘Mystic Isle’, a VR game used for homework practice, as an intervention tool was confirmed. A summary and limitations of this study are presented in the Table (Table 3 and Table 4).

Metcalfe et al. (2019) conducted a single-subject study for two outpatients with stroke (A: 55-year-old female, cerebral infarction, left hemiplegia, onset period 144 months; B: 57-year-old male, cerebral infarction, onset period 36 months) [28]. The study was designed with a total of 16 sessions including baseline (four sessions), intervention (six sessions), and postintervention phases (four sessions).

The LB intervention was conducted by an occupational therapist, and the intervention phase that only conducted LB consisted of daily exercise, functional component movements, and hierarchy tasks. The homework practice conducted separately during the intervention period consisted of daily exercise and functional component movements, which were used in the intervention, and separate homework designed to use large amplitude in daily life, to be performed at home. In addition, the Canadian Occupational Performance Measure (COPM) was used to select three goal occupations that each subject individually desired, and only one occupation was included in the LB.

The evaluation was conducted every 16 sessions or before/after the intervention phase. In the average score of the results evaluated every 16 sessions, most COPM performance and satisfaction improved in all subjects after the intervention phase. However, performance (pre: 3.04, post: 5.1) and satisfaction (pre: 2.18, post: 5) in the occupation included in LB showed more improved results than performance (pre: 3.79, post: 5.05) and satisfaction (pre: 3.63, post: 5.2) of the occupation to which LB was not applied. Moreover, in the Performance Quality Rating Scale-Operational Definition (PQRS-OD, total score: 10) to determine the completeness and quality of occupational performance designated as COPM in order to compare the effects before and after training, both subjects showed more improvements in the occupation to which LB was applied. In addition, in the Rating of Everyday Arm-Use in the Community and Home (REACH) to determine the frequency of use of upper extremity, the frequency of use was maintained, and there was no significant difference in the Chedoke Arm and Hand Activity Inventory-13 (CAHAI-13) to determine the function of the arm and hand. A summary and limitations of this study are presented in the Table (Table 3 and Table 4).

Jeong and Hong (2020) conducted a case study for two inpatients with stroke (A: 55-year-old male, cerebral infarction, left hemiplegia, onset period 10 months/B: 55-year-old male, cerebral hemorrhage, right hemiplegia, onset period 66 months) [29].

The LB intervention was conducted by an occupational therapist over a total of 16 sessions, consisting of daily exercises, functional component tasks, hierarchy tasks, and BIG walking. The intensity of the intervention was set to 80% of the maximum exercise volume for each subject.

The evaluation was conducted twice in total: pre-intervention and post-intervention. In the comparison of pre- and post-intervention, there was no change on the affected side (Lt side) of subject A in the Manual Function Test (MFT) that evaluates upper extremity function, and the total score on the affected side (Rt side) of subject B improved by 3 points (proximal 1, distal 2) from 26 to 29 points. In the Functional Reaching Test (FRT), a dynamic balance assessment, both subjects showed improvement on the less affected side as well as the affected side (subject A (Rt/Lt): 6.5 cm (pre: 23.6, post: 30.1)/4.2 cm (pre: 24.3, post: 28.5); subject B (Rt/Lt): 3.8 cm (pre: 10.1, post: 13.9)/8.2 cm (pre: 12.3, post: 20.5)). They also showed improvement in the Berg Balance Scale (BBS, total score: 56), a balance assessment (subject A: pre: 47, post: 50; subject B: pre: 34, post: 40) and the Time Up and Go (TUG), a gait and balance assessment (subject A: pre: 8.71, post: 7.80; subject B: pre: 28.28, post: 19.86). Lastly, in the COPM for evaluating occupational performance, the subject A’s average performance and satisfaction improved by 4 and 4.5 points, respectively, while the subject B’s averages improved by 1 and 3 points, respectively. A summary and limitations of this study are presented in the Table (Table 3 and Table 4).

Proffitt et al. (2021) conducted a waitlist cross-over study to investigate the intervention feasibility through LB in five outpatients with stroke (A: 56-year-old male, left hemiplegia, onset period 31 months/B: 65-year-old female, right hemiplegia, onset period 14 months/C: 42-year-old male, left hemiplegia, onset period 89 months/D: 49-year-old male, right hemiplegia, onset period 17 months/E: 68-year-old female, right hemiplegia, onset period 8 months/all subjects’ premorbid dominant hand was right hand) [30]. As for study design, a pre-evaluation without group division was performed at baseline (T1) before training, and LB was performed only on one group (*n* = 3) for the first 4 weeks (T2) after the start of training. Then, LB was applied only to the remaining group (*n* = 2) for the following 4 weeks (T3). All groups were not given separate rehabilitation therapy during the non-intervention period.

The LB intervention was conducted by an occupational therapist over a total of 16 sessions, consisting of daily exercises, functional component movements, and hierarchy tasks. Additionally, homework practices conducted separately during the intervention period were designed to use the daily exercises and functional component movements, which were used in the intervention, and large movements in daily life. They were conducted at home on days when the LB was received (one session) and days when the LB was not received (two sessions), respectively. The intensity of the intervention was set to 7 points or higher on a 10-point self-report scale.

The evaluation was conducted three times in total, from T1 to T3. Compared to the baseline T1, most subjects showed improvement in the performance and satisfaction of COPM and the performance time and score of WMFT, which evaluates upper extremity function, during T2 and T3. Furthermore, more improvement was shown in the intervention period than in the non-intervention period. Three out of five subjects showed improvement in the independence, safety, and adequacy scores of PASS, which evaluates activities of daily living. Finally, all subjects reported positive changes in anxiety, depression, social roles, and activity participation ability in the National Institutes of Health Patient Reported Outcomes Measurement Information System-43 (PROMIS-43), which evaluates physical, mental, and social health status. A summary and limitations of this study are presented in the Table (Table 3 and Table 4).

### 3.2. Other LB Studies on Neurological Diseases—4 Articles

Brown (2019) conducted a case report to examine the effects of LB program intervention for one outpatient with progressive supranuclear palsy (PSP) (69-year-old male, diagnosed with PSP 3 months ago and Parkinson’s disease 2 years ago) [31].

The LB intervention was conducted by a physical therapist over a total of nine sessions, consisting of daily exercises, functional component movements, hierarchy tasks, and BIG walking. Even on days when there was no treatment, similar exercises were performed three to four times a week at home through a guardian. The exercise was conducted at an intensity perceived as 75–85% of effort using the Borg RPE (Rating of Perceived Exertion) scale.

The evaluation was conducted twice in total: pre-intervention and post-intervention. The Functional Gait Assessment (FGA), which examines postural stability in various gait tasks, showed a 2-point improvement (post: 19/30). However, the Berg Balance Scale (BBS), which evaluates the static and dynamic balance and the risk of fall, showed a 2-point decrease, indicating worse balance (post: 31/56). The 6 Minute Walk Test (6MWT), which examines the distance and average speed of walking for 6 min, also showed a decrease in both walking speed and distance, with 89 m in the distance and 0.25 m/s in the speed (post: 350.5 m, 0.97 m/s). In addition, the 5 Times Sit to Stand (5TSTS), which is a test used to quantify the functional lower extremity muscle strength, showed a 2.5 s increase in total performance time (post: 34 s). This patient, who was recently diagnosed with PSP, showed improved walking stability and walking speed, but had mixed results, showing a decrease in static and dynamic balance, functional lower extremity muscle strength, and aerobic capacity. A summary and limitations of this study are presented in the Table (Table 5 and Table 6).

Fillmore et al. (2020) performed the world’s first case report on the LB program intervention for one outpatient with Idiopathic Normal Pressure Hydrocephalus (INPH) (62-year-old male, diagnosed with INPH 16 years ago) [32].

The LB intervention was conducted by a physical therapist, and 10 out of 12 sessions were conducted. BIG Walking was included in the functional component movements due to the subject’s distractibility. The home exercise training program was performed once a day on days with LB and twice a day on days without LB.

The evaluation was conducted four times in total: pre-intervention (T1), post-intervention (T2), 4 months after the end (T3, follow-up), and 7.5 months after the end (T4, tune up session) (however, getting off the floor test was not conducted at T3). The tune-up session refers to a session that provides feedback on motivation/exercise patterns after the completion of the LB program. The T1-T4 results of the Berg Balance Scale (BBS, total score: 56), which examines the sense of balance, showed changes of 34, 54, 47, and 53 points, respectively. The T1-T4 results of the Activities-Specific Balance and Confidence (ABC, total score: 100%), which measures confidence in walking activities, showed changes of 36.3%, 82.2%, 70.3%, and 69.4%, respectively. Additionally, the improvements in the BBS and ABC scales exceeded the minimal detectable changes (MDC) after the intervention, showing that the LB intervention was effective in functional improvement. The Time Up and Go (TUG), which evaluates balance, gait ability, and risk of fall by measuring 3 m round trip time, showed functional improvement by decreasing to 9.07, 10.19, 8.5, and 7.59 s in the results of T1-T4, respectively. In TUG cognition, which assigns a cognitive task during the TUG evaluation, the functional improvement was shown by decreasing to 10.07, 10.29, 8.3, and 8.56 s, and TUG manual, which assigns a hand movement task, showed a decrease to 10.03, 9.24, 8.15, and 19.44 s, but then increased again in the tune-up session. The 5 Times Sit to Stand test (5TSTS), which evaluates functional lower extremity muscle strength, showed final functional improvement with 14.12, 15.01, 13.88, and 10.73 s. The getting off the floor test (T1, T2, T4), which measures the time to get up from the floor as quickly as possible in a safe way, showed changes to 8.26, 5.56, and 8.96 s. However, the TUG, TUG cognitive and manual, 5TSTS test, and getting off the floor test did not exceed the MDC, indicating that the LB intervention effect was not high. In addition, the LB Follow-Up Questions, which were used to evaluate the subjects’ subjective reports on mobility, were conducted immediately after the 4-month follow-up after the end of the intervention. Overall, the subjects showed the greatest improvement immediately after the intervention. It was reported after 4 months that the results were worse than immediately after the intervention but not worse than before the intervention. Based on this, the potential of the LB program for INPH patients with hypokinesia and bradykinesia could be confirmed. A summary and limitations of this study are presented in the Table (Table 5 and Table 6).

Hoyman (2022) conducted a case report on the LB program intervention for one outpatient with Huntington’s disease (48-year-old male, diagnosed 6 years ago) [33].

The LB intervention was conducted by a physical therapist for a total of 8 weeks and consisted of daily exercises, functional component movements, hierarchy tasks, and BIG Walking. It was initially conducted 45 min a day, 3 times a week, and gradually decreased to 2 times a week and finally 1 time a week. The home program was conducted from the third week.

The evaluation consisted of initial evaluation (T1) and re-evaluations of 4 weeks after (T2) and 8 weeks after at discharge (T3), and the 6-min walking test (6MWT), Timed Up and Go (TUG), gait assessment, karaoke stepping, and motor coordination test were performed. The 6MWT was 1315 feet in the initial evaluation (T1), which was within the normal range, so it was not re-evaluated further. The TUG scores (T1, T2, T3), which warn of the risk of fall when the score is 12 or higher, were 11.99, 7.21, and 7.75 s, respectively, showing a total decrease of 4.24 s (the minimum clinically significant change is 3.4 s). In the gait assessment, the patient’s heel strike increased and path deviation decreased after 4 weeks (T2) and 8 weeks (T3) compared to the initial evaluation (T1), but a wide base of support was still maintained. Karaoke stepping was almost impossible to perform in the initial evaluation, but after 4 weeks, the patient was able to perform three consecutive steps of karaoke stepping well, and around 8 weeks, the patient was able to perform five consecutive steps of karaoke stepping in both directions when there was a demonstration and verbal instructions. Motor coordination test (measured only at T1 and T2) was measured by performing rapid alternating movements such as supination/pronation and finger to nose in the upper extremity and dorsiflexion/plantarflexion, heel to shin test, and toe to target in the lower extremity. Overall, coordination of both the upper and lower extremities improved. The patient was administered antipsychotic medication (Risperdal) at the third week of the intervention. After this concurrent drug treatment, the patient showed functional improvement in all outcome measures after 1 month, but the functional improvement stagnated thereafter. Although there is no treatment that can cure Huntington’s disease, it was confirmed that LB intervention has the potential to alleviate the symptoms of the disease. A summary and limitations of this study are presented in the Table (Table 5 and Table 6).

Hirakawa et al. (2023) conducted a case report on the LB program intervention for one outpatient with Progressive Supranuclear Palsy (PSP) (74-year-old male, former self-employed, unemployed currently, diagnosed with PSP 1 year ago) [34].

The LB intervention was conducted by a physical therapist over a total of 16 sessions, consisting of daily exercises, functional component movements, hierarchy tasks, and BIG Walking. In all parts of the 1-h treatment, the therapist monitored whether the exercise load was maintained at a high intensity within the range of 7 to 8 points out of 10 on the modified Borg scale. The home program was conducted a total of 20 sessions.

The evaluation was conducted one day before the start of the intervention and the day after the completion of the LB intervention. Before starting the LB intervention, the participant was only receiving drug treatment and did not receive physical therapy. Progressive Supranuclear Palsy Rating Scale (PSPRS), Unified Parkinson’s Disease Rating Scale Part 3 (UPDRS Part 3), and BBS were used to evaluate limb movement and gait ability. In addition, festinating gait with quick walking pace was evaluated using the gait subsections of UPDRS part 3 and the 10 Meter Walk Test (10MWT). Since it was to return to the pace of the participant before the onset of PSP, a decrease in gait speed was defined as improvement. Finally, the achievement of specific goals was evaluated through a Q&A session.

After the intervention, in the results of the PSPRS and UPDRS, where a decrease means an improvement, the disability of the limb item (total score: 16) in the PSPRS sub-items improved (pre: 9, post: 5), and the disability of the PSPRS gait item (total score: 20) also improved (pre: 8, post: 6). The UPDRS Part 3 score (total score: 56) also decreased, indicating a decrease in motor disability (pre: 30, post: 21). The BBS score (total score: 56), which evaluates balance ability, increased after the intervention, indicating an improvement in the balance ability (pre: 45, post: 50). The gait subsection score of the UPDRS Part 3 for evaluating festinating gait with quick walking pace (total score: 4) decreased (pre: 2, post: 1). This is a result of an improvement in festinating gait. Also, the 10MWT was reduced (pre: 1.65 m/s, post: 1.10 m/s), which can be interpreted as an improvement in festinating gait. After 1 week of intervention, the participant was able to independently resume walking at the original pace, but the gait was unsteady and could not be maintained for more than a few meters. However, after 4 weeks, the participant was able to independently maintain the original pace while speaking. Additionally, in the last Q&A, he answered, “I can now walk at a comfortable pace, maintain that pace easily, and match my wife’s pace.” Based on this, the potential of LB as an intervention tool for symptoms of PSP patients was reported for the first time. A summary and limitations of this study are presented in the Table (Table 5 and Table 6).

## 4. Discussion

This study aimed to investigate the effectiveness of LB and its potential as an intervention tool for patients with neurological diseases. Accordingly, a total of eight LB studies were closely analyzed.

Looking at the effectiveness and assessment tools of LB, studies on stroke reported the greatest improvements in the areas of upper function (four studies) [27,28,29,30], occupational function (four studies) [27,28,29,30], and activities of daily living (two studies) [27,30]. As for assessment tools, COPM (four studies) [27,28,29,30] was used the most to examine occupational function, followed by WMFT for upper function (two studies) [27,30] and PASS for activities of daily living (two studies) [27,30]. In addition, tools for spasticity, upper extremity use rate, balance, gait, QOL, and POQ were used (Table 3). It is believed that the main reason why tools from the occupational therapy area were used the most for assessment is that occupational therapists conducted the studies. In studies on other diagnoses, improvements were reported in the areas of gait (four studies) [31,32,33,34], balance (three studies) [31,32,33], and motor function (two studies) [32,34]. Looking at assessment tools, the most commonly used tool was BBS for balance (three studies) [31,32,34], followed by TUG for gait (two studies) [32,33], 5 Time Sit to Stand for muscle strength (5TSTS, two studies) [31,32], and Follow-Up Questions (two studies) [32,34]. In addition, tools for coordination, etc. were used (Table 5). The reason why many of these assessment tools, which are in the area of physical therapy that focused on gait and lower extremity function, were used is thought to be because the studies were conducted by physical therapists. Moreover, most of the eight studies mainly used quantitative assessments, and only three studies [27,28,34] dealt with both quantitative and qualitative assessments. Since LB training focuses on the subject’s own movement awareness and amplitude recalibration in the therapist’s modeling that emphasizes large amplitude [18,21], assessments focusing on qualitative changes will also be necessary. Additionally, for accurate quantitative and qualitative assessments on physical functions after LB intervention for patients with neurological diseases, it is thought to be important to use comprehensive and valid assessment tools, not those limited to the researcher’s specialty.

LB program compromises daily exercise, functional component movements, hierarchy tasks, BIG walking, and a home program. It is recommended that training be performed at maximum intensity for consecutive 4 days a week, a total of 16 sessions for 4 weeks. Additionally, the home program protocol is to perform once on days with LB intervention and twice on days without LB intervention. All studies conducted the intervention in a person-to-person manner. However, among the eight studies, there were studies that performed the training 3 times a week for 3 weeks [31], 3 times a week for 4 weeks [32], or reduced the training frequency from 3 times a week to 1 time for 8 weeks [33]. In studies targeting stroke, there were studies that had a good basic program structure but did not mention the intensity of the intervention [27,28] or BIG walking [28,30], did not conduct a home program [29], or did not mention it in detail [28]. In addition, there were studies that did not mention consecutive 4 days [28,29], indicating that they did not follow the rule specified in the protocol. In studies targeting diseases other than stroke, there were studies that had no intensity of the intervention [33], included BIG walking in functional component movements [32], or did not differentially apply the number of home program implementation depending on the presence or absence of LB [31,32,33,34]. Therefore, it is determined that there is a need to follow the protocol of LB in future studies.

In this study, we first summarized four studies on stroke patients. The COPM used in four stroke studies all showed improved outcomes [27,28,29,30]. This is due to the correlation between COPM goal setting and LB training elements, functional component movements, and hierarchy tasks [35]. However, it is regrettable that only four studies on stroke patients were conducted and that the studies were designed with small samples. Moreover, in the case of hemiplegic patients, it is thought that a detailed description on the dominant hand and whether the improvement was on the left or right hand is necessary. Next, we summarized four studies on neurological diseases other than stroke. First, LB applied to PSP patients in this study showed improvements in lower extremity muscle strength, limb movement, gait, and balance [31,34]. It is known that approximately 4% of Parkinson’s disease patients suffer from PSP [36]. Compared with Parkinson’s disease, PSP patients are characterized by rapid disease progression in the early stage, no or weak response to dopaminergic drugs, postural imbalance, falls, dysphagia, and dysarthria [37]. Since bradykinesia may be the only initial sign of basal ganglia dysfunction in the early stage of PSP, the application of the LB program in the early stage is considered important. Next, there is strong treatment evidence that moderate aerobic exercise combined with upper and lower body strengthening exercises can help improve motor function in Huntington’s disease and that gait training can improve the spatiotemporal ability of gait [38]. It is thought that the LB protocol including these training methods was effective for the Huntington’s disease patient since it was performed in combination with patient education, coordination training, and balance training. Finally, INPH patients are characterized by decreased gait velocity, shortened stride length, shuffling gait, freezing of gait, decreased postural response, difficulty in transitional movements, wide step width, external rotation of the legs, and limited step height [39]. In this study, which confirmed the effect of LB on INPH patients, improvements in balance and confidence in balance were observed. It is thought that the sensory recalibration principle of perceived movement and amplitude training implemented through high-intensity and high-effort training, which are characteristics of LB, contributed to the improvement of sensory attention and body awareness, thereby helping to improve the balance and confidence of INPH patients [40].

The major limitations of these studies are as follows: (1) small sample size [27,28,29,30,31,32,33,34], (2) insufficient compliance with the LB protocol [28,29,30,31,32,33], (3) failure to mention the objective intensity when performing LB [27,29,32,33], and (4) lack of mention of premorbid dominant hand in hemiplegic stroke patients [28,29]. Moreover, since LB is a relatively new treatment approach for Parkinson’s disease, it has been applied to other neurological diseases in a limited way. However, in most of the studies included in this study, LB had a positive effect on improving physical function and overall motor control in subjects with neurological diseases other than Parkinson’s disease, indicating a high need for follow-up studies. In future studies, it is expected that protocols for applying LB to group therapy will be developed to solve the problem of small sample size in all studies, and that studies taking into account the aforementioned limitations will be conducted. Nevertheless, it is meaningful that this is the first review of studies that conducted LB on patients with neurological diseases, and it is thought that more diverse studies targeting more diverse patients will be needed in the future.

## 5. Conclusions

In this study, a total of eight studies were reviewed to determine the effectiveness of LB on patients with neurological diseases. LB had a positive effect on improving physical function and overall motor control in patients with stoke, progressive supranuclear palsy, Huntington’s disease, and idiopathic normal pressure hydrocephalus, which served as an opportunity to confirm its therapeutic potential. Follow-up studies should be conducted considering expanding the sample size to a wider range of patients, compliance with the LB protocol, standardization of intervention intensity, use of comprehensive and valid assessment tools, and follow-up observation for the effects after the intervention.

## Figures and Tables

**Table 1:** Composition of standard LSVT-BIG Program Sessions.

Session (Time)	Session Contents	Details
1st half (30 min or more)	Daily Exercise	1. Floor to ceiling (seated) eight repetitions (10 s hold)
		2. Side to side (seated) eight repetitions (10 s hold)
		3. Forward step and reach (standing) eight repetitions each leg
		4. Sideways step and reach (standing) eight repetitions each side
		5. Backward step and reach (standing) eight repetitions each leg
		6. Forward rock and reach (standing) 10 repetitions each leg
		7. Sideways rock and reach (standing) 10 repetitions each side
2nd half (30 min. or less)	Functional Component movements	Five everyday tasks that the subject wants to perform more successfully—five repetition each
	Hierarchy Tasks	Activities that are divided into stages for more difficult movements targeting the movements that have been resolved among daily life or functional component movements
	BIG Walking	Walking during various distances and time limits with large strides
Homework practice		Treatment day: one time for 5–10 min Nontreatment day: two time for 10–15 min

With respect to LB, various studies on Parkinson’s disease and positive outcomes have been reported [17,18,19,20,21,24,25,26]. Most studies have targeted Parkinson’s disease patients, and review studies on its effectiveness have also targeted only Parkinson’s disease patients [16,24,25,26]. On the other hand, there have been no review studies on the effectiveness of LB for neurological diseases other than Parkinson’s disease. Therefore, this study aims to examine studies on LB for diseases other than Parkinson’s disease, conduct a review study on its effectiveness, and identify its potential as an intervention tool.

**Table 2:** Electronic databases used in the search, search keywords, and inclusion criteria.

Electronic Databases	Google Scholar, PubMed, ScienceDirect
Search keyword	Neurological disease, LSVT-BIG, Treatment or Rehabilitation, Intervention, Therapy
Criteria for inclusion	Written in English; Performed LSVT-BIG; Available in Full text; Published in journals

**Table 3:** Intervention Summaries—Four stroke studies.

Authors (Year)	Licenses of Trainer	Design/Subject (in/Outpatient)	Diagnosis		Evaluation				
				Spasticity	Upper-Extremity Function/Use Rate	Occupational Function/POQ	Balance/Gait	ADL/QOL	Health Condition
Proffitt et al. (2018) [27]	OT	Case study/*n* = 1 (out)	Ischemic stroke	MAS	WMFT	COPM		PASS/SS-QOL	
Metcalfe et al. (2019) [28]	OT	Single-subject design /*n* = 2 (out)	A: Rt. Side ischemic stroke B: Stroke		CAHAI-13/REACH	COPM/PQRS-OD			
Jeong & Hong (2020) [29]	OT	Case study/*n* = 2 (in)	A: Infarction B: Hemorrhage		MFT	COPM,	BBS, FRT/TUG		
Proffitt et al. (2021) [30]	OT	Waitlist crossover design/*n* = 5 (out)	Stroke		WMFT	COPM		PASS	PROMISE-43

ADL: Activities of Daily Living, QOL: Quality Of Life, POQ: Performance Of Quality, OT: Occupational Therapist, MAS: Modified Ashworth Scale, WMFT: Wolf Motor Function Test, COPM: Canadian Occupational Performance Measure, PASS: Performance Assessment of Self-Care Skills, SS-QOL: Stroke-Specific Quality of Life scale, CAHAI-13: Chedoke Arm and Hand Activity Inventory-13, REACH: Rating of Everyday Arm-use in the Community and Home, PQRS-OD: Performance Quality Rating Scale-Operational Definition, MFT: Manual Function Test, BBS: Berg Balance Scale, FRT: Functional Reaching Test, TUG: Time Up and GO, PROMIS-43: Patient-Reported Outcomes Measurement Information System-43.

**Table 4:** Intervention Results—Four stroke studies.

Authors (Year)	Intervention					Result	Limitation
	DE	FCM	HT	BW	HP		
Proffitt et al. (2018) [27]	O	O	O	O	O	Improvement in all tests (MAS, WMFT, COPM, PASS, SS-QOL)	1. Small sample size 2. Only 50% of the planned homework practice was performed. 3. The items of the five tasks in the PASS results are not mentioned. 4. The intensity of the LB intervention is not mentioned. 5. It is impossible to identify which component of the LB contributed most to the functional improvement.
	PTP type, 1-h session/day, consecutive 4 days/week for 4 weeks				1-h session/day (treatment on days), 2-h session/day (nontreatment on days) for 4 weeks		
Metcalfe et al. (2019) [28]	O	O	O	X	O	REACH: Maintained PQRS-OD: Improvement in some items COPM: Improvement CAHAI-13: No significant difference	1. Small sample size and no mention of premorbid dominant hand. 2. It is impossible to identify which component of the intervention contributed to the improvement of occupational performance. 3. For the LB protocol, BIG Walking, only four times a week is mentioned, and there is no mention of consecutive four times a week as mentioned in the protocol. 4. No clear description on homework practice and intervention intensity. 5. Occupational performance evaluation was conducted only through the subjective assessment COPM.
	PTP type, 1-h session/day, 4 days/week for 4 weeks				Not mentioned in detail		
Jeong & Hong (2020) [29]	O	O	O	O	X	Improvement in MFT (Only case B), FRT, BBS, TUG, COPM	1. Small sample size. 2. No mention of the subjects’ premorbid dominant hand. 3. Lack of prior mention of the degree of recovery of stroke patients to whom LB can be applied, resulting in differences in functional recovery for each subject. 4. In performing LB, only four times a week was mentioned, making it unclear whether it would be implemented consecutive four times a week as mentioned in the protocol. 5. No homework practice. 6. It is difficult to determine the effect of LB alone since other rehabilitation therapies were performed simultaneously with LB application. 7. It is impossible to identify which component of the LB contributed most to the functional improvement. 8. There is a difference in the number of stroke occurrences and the frequency of rehabilitation therapy other than LB in only two subjects.
	PTP type, 1-h session/day, 4 days/week for 4 weeks The intensity of the intervention was 80% of the maximum exercise volume for each subject.						
Proffitt et al. (2021) [30]	O	O	O	X	O	Improvement in all tests (COPM, WMFT, PASS, PROMIS-43)	1. Small sample size. 2. There is no pre/post comparison of MAS, and BIG Walking is excluded from the basic structure. 3. No left-right distinction of improved results of the subject in WMFT. 4. It is impossible to identify which component of the LB contributed most to the functional improvement. 5. Not all 16 sessions could be performed due to accessibility issues of the intervention site.
	PTP type, 1-h session/day, consecutive 4 days/week for 4 weeks The intensity of the intervention was a score of 7 or higher on a 10-point self-report scale.				20–40 min session/day for 4 weeks (once on treatment days and twice on nontreatment days)		

DE: Daily Exercise, FCM: functional component movements, HT: hierarchy tasks, BW: BIG walking, HP: Homework practice, PTP: person to person, ADL: Activities of Daily Living, QOL: Quality Of Life, POQ: Performance Of Quality MAS: the Modified Ashworth Scale, WMFT: Wolf Motor Function Test, COPM: Canadian Occupational Performance Measure, PASS: Performance Assessment of Self-Care Skills, SS-QOL: the Stroke-Specific Quality of Life scale, REACH: Rating of Everyday Arm-use in the Community and Home, PQRS-OD: Performance Quality Rating Scale-Operational Definition, CAHAI-13: the Chedoke Arm and Hand Activity Inventory-13, MFT: Manual Function Test, BBS: Berg Balance Scale, FRT: Functional Reaching Test, TUG: Time Up and GO, PROMIS-43: The National Institutes of Health Patient Reported Outcomes Measurement Information System-43.

**Table 5:** Intervention Summaries—four studies on neurological diseases other than stroke.

Authors (Year)	Licenses of Trainer	Design/Subject (in/Outpatient)	Diagnosis	Evaluation					
				Muscle Strength	Balance	Motor	Gait	Coordination	Follow-Up Questions
Brown (2019) [31]	PT	Case report/*n* = 1 (out)	PSP	5TSTS	BBS		FGA 6MWT		
Fillmore (2020) [32]	PT	Case report/*n* = 1 (out)	INPH	5TSTS	BBS ABC	Getting off the floor	TUG TUG- cognition TUG- manual		Follow-Up Questions
Hoyman (2022) [33]	PT	Case report/*n* = 1 (out)	Huntington’s Disease				TUG gait assessment karaoke stepping	coordination	
Hirakawa (2023) [34]	PT	Case report/*n* = 1 (out)	PSP		BBS	PSPRS-limb PSPRS-gait UPDRS Part3	10MWT		Follow-Up Questions

**Table 6:** Intervention Results—four studies on neurological diseases other than stroke.

Authors (Year)	Intervention					Result	Limitation
	DE	FCM	HT	BW	HP		
Brown (2019) [31]	O	O	O	O	O	FGA: Improvement BBS, 6MWT, 5TSTS: Deterioration	1. Small sample size. 2. It is clinically difficult to provide effective interventions to patients with progressive diseases such as PSP and improve the scores. 3. It is difficult to judge the intervention effect when the patient’s function is deteriorating to the extent that it is impossible to respond to treatment intervention due to the progression of the disease. 4. The treatment was only performed three times a week, and it is impossible to know whether it was performed consecutively.
	PTP type, 1-h session/day, 3 days/week for 3 weeks The intensity of the intervention was 75~85% of the subject’s maximum exercise volume.				1-h session/day, 3~4 days/week for 3 weeks		
Fillmore (2020) [32]	O	O	O		O	ABC, BBS: Improvement TUG, TUG cognitive and manual, 5TSTS test, Getting off the floor: No difference Subjective assessment: Improvement	1. There is a limitation in generalizing the observation results since it was conducted for only one participant. 2. It is difficult to directly link the improvement in the result to the LB intervention and its effect since there is no previous physical therapy experience. 3. There is a limitation in interpreting the intervention results only with the MDC results. 4. The program could not be performed for a sufficiently long period due to the subject’s cognitive impairment. 5. The LB program needs to be operated according to the cognitive status and learning ability of each patient. 6. The intervention was only performed on consecutive 3 days a week, and no participation was made twice during the entire schedule. 7. There is no objective mention of the intensity of the intervention (standardized maximum daily exercise volume).
	PTP type, 1.5-h session/day, consecutive 3 days/week for 4 weeks				1.5-h 2 sessions/day 3 days/week for 4 weeks		
Hoyman (2022) [33]	O	O	O	O	O	TUG: Improvement Gait assessment, karaoke stepping: Improvement in some items Coordination: Improvement	1. Small sample size. 2. A home exercise program was implemented from the third week. 3. Initially, treatment was performed three times a week, but the number of treatments was reduced to one time a week after symptom improvement. 4. Since most physical therapy interventions were performed together with LB exercise, it is difficult to compare LB exercise with other physical therapy interventions performed for Huntington’s disease. 5. LB intervention was performed on the subject in the intermediate stage of Huntington’s disease symptoms. 6. No specific mention of intervention intensity. 7. After administering antipsychotic medication at the third week, all outcome measures showed functional improvement but stagnated thereafter, so the contribution of the medication can be considered.
	PTP type, 45-min session/day, 1~3 days/week for 8 weeks				Home exercise from the third week 5 days/week for 6 weeks		
Hirakawa (2023) [34]	O	O	O	O	O	Improvement in all tests (PSPRS, UPDRS, BBS, 10 MWT, Follow-Up Questions)	1. The results cannot be generalized due to the small sample size. 2. Since PSP gait abnormalities vary from patient to patient, additional research is needed to examine the generalizability of LB to various PSP patients. 3. It is difficult to identify what made the effect as medications were administered before implementation of LB.
	PTP type, 1-h session/day, consecutive 4 days/week for 4 weeks The intensity of the intervention was 70–80% of the subject’s maximum exercise volume.				(1-h session/day, 5 days/week for 4 weeks		

DE: Daily Exercise, FCM: Functional Component Movements, HT: Hierarchy Tasks, BW: BIG Walking, HP: Homework Practice, PTP: Person to Person, FGA: Functional Gait Assessment, BBS: Berg Balance Scale, 6MWT: 6 Minute Walk Test, 5TSTS: 5 Times Sit to Stand, ABC: Activities-Specific Balance and Confidence, TUG: Timed Up and Go, PSPRS: Progressive Supranuclear Palsy Rating Scale, UPDRS: Unified Parkinson’s Disease Rating Scale. 10MWT: 10 Meter Walk Test, PSP: Progressive Supranuclear Palsy, INPH: Idiopathic Normal Pressure Hydrocephalus, MDC: Minimal Detectable Change.

## Abbreviations

LB	Lee Silverman Voice Treatment-BIG
LSVT	Lee Silverman Voice Treatment
TBI	Traumatic Brain Injury
VR	Virtual Reality
ADL	Activities of Daily Living
QOL	Quality Of Life
POQ	Performance Of Quality
OT	Occupational Therapist
MAS	Modified Ashworth Scale
WMFT	Wolf Motor Function Test
COPM	Canadian Occupational Performance Measure
PASS	Performance Assessment of Self-Care Skills
SS-QOL	Stroke-Specific Quality of Life scale
CAHAI-13	Chedoke Arm and Hand Activity Inventory-13
REACH	Rating of Everyday Arm-use in the Community and Home
PQRS-OD	Performance Quality Rating Scale-Operational Definition
MFT	Manual Function Test
BBS	Berg Balance Scale
FRT	Functional Reaching Test
TUG	Timed Up and GO
PROMIS-43	Patient-Reported Outcomes Measurement Information System-43
DE	Daily Exercise
FCM	Functional Component Movements
HT	Hierarchy Tasks
BW	BIG Walking
HP	Homework Practice
PTP	Person to Person
PT	Physical Therapist
PSP	Progressive Supranuclear Palsy
INPH	Idiopathic Normal Pressure Hydrocephalus
FGA	Functional Gait Assessment
6MWT	6-Minute Walk Test
5TSTS	5-Time Sit to Stand
ABC	Activities-Specific Balance and Confidence
PSPRS	Progressive Supranuclear Palsy Rating Scale
UPDRS:	Unified Parkinson’s Disease Rating Scale
10MWT	10-Meter Walk Test
